# Cardiovascular risk and statin prescription in primary care: an observational study, São Leopoldo, 2018-2021

**DOI:** 10.1590/S2237-96222025v34e20240432.en

**Published:** 2025-10-20

**Authors:** Vania Celina Dezoti Micheletti, Roger dos Santos Rosa, Thiago Dipp, Luise Peter da Silva, Paula Dal Bó Campagnolo, Luís Richard Mercaus, Jéssica Rosiane de Brito, Maria Eduarda Moutinho Bonin

**Affiliations:** 1 Universidade do Vale do Rio do Sinos, Programa de Pós-Graduação em Saúde Coletiva, São Leopoldo, RS, Brazil; 2 Universidade Federal do Rio Grande do Sul, Faculdade de Medicina, Porto Alegre, RS, Brazil; 3 Universidade Federal do Rio Grande do Sul, Programa de Pós-Graduação em Saúde Coletiva, Porto Alegre, RS, Brazil; 4 Universidade do Vale do Rio do Sinos, Mestrado Profissional em Alimentos, Nutrição e Saúde, São Leopoldo, RS, Brazil; 5 Universidade do Vale do Rio do Sinos, Programa de Residência Multiprofissional em Atenção Básica, São Leopoldo, RS, Brazil; 6 Universidade Luterana do Brasil, Programa de Residência Multiprofissional de Saúde Comunitária, Canoas, RS, Brazil

**Keywords:** Primary Prevention, Primary Health Care, Cardiovascular Diseases, Hypolipidemic Agents, Cholesterol, Prevención primaria, Atención primaria de salud, Enfermedades cardiovasculares, Hipolipemiantes, Colesterol

## Abstract

**Objectives:**

To present the cardiovascular risk strata based on the Global HEARTS Initiative calculator and analyze statin prescription among users of Primary Health Care in São Leopoldo, Rio Grande do Sul.

**Methods:**

This was a cross-sectional observational study including users aged 18 years or older listed in the Cardiovascular Risk Operational Report. Categorical data were presented as absolute and relative frequencies and 95% confidence intervals (95%CI). Associations between categorical variables were analyzed using the chi-square test or Fisher’s exact test, with statistical significance defined as p-value≤0.050.

**Results:**

It was possible to calculate the cardiovascular risk of 2,199 users. Most users presented low (41.4%; 95%CI 39.3; 43.5) or moderate risk (38.0%; 95%CI 36.0; 40.1), while 19.6% (95%CI 18.0; 21.3) had high risk and 1.0% (95%CI 0.6; 1.4) had very high risk. Statin prescription was recorded in 29.5% (95%CI 27.6; 31.4) of the medical records. There was a statistically significant association (p-value<0.001) between cardiovascular risk and statin prescription, with higher prescription frequency among high-risk users (41.2%).

**Conclusion:**

Most Primary Health Care users in São Leopoldo had low to moderate cardiovascular risk. The prescription of statins was consistent with clinical guidelines, considering cardiovascular risk in decision-making; however, it was reached by less than one-third of the overall sample.

## Resumen

**Objetivos:**

Presentar los estratos de riesgo cardiovascular según la calculadora de la iniciativa HEARTS y analizar la prescripción de estatinas a los usuarios de la Atención Primaria de Salud de São Leopoldo, Rio Grande do Sul.

**Métodos:**

Se trata de un estudio observacional transversal que incluyó a usuarios con una edad mínima de 18 años presentes en el Informe Operativo de Riesgo Cardiovascular. Los datos categóricos se presentaron en frecuencias absolutas y relativas y en intervalos de confianza del 95 % (IC95 %). Para analizar las asociaciones entre las variables categóricas, se utilizó la prueba de chi cuadrado o la prueba exacta de Fisher con significación estadística si el valor p era ≤0,050.

**Resultados:**

Se pudo calcular el riesgo cardiovascular de 2199 usuarios. La mayoría de los usuarios presentaban un riesgo bajo (41,4 %; IC95 % 39,3; 43,5) o moderado (38,0 %; IC del 95 %: 36,0; 40,1), mientras que el 19,6 % (IC del 95 %: 18,0; 21,3) presentaban un riesgo alto y el 1,0 % (IC del 95 %: 0,6; 1,4), un riesgo muy alto. El registro de la prescripción de estatinas figuraba en el 29,5 % (IC95 % 27,6; 31,4) de los historiales clínicos. Se observó una asociación significativa (valor p<0,001) entre el registro de la prescripción de estatinas y el riesgo cardiovascular, con mayor frecuencia en usuarios con riesgo alto (41,2 %).

**Conclusión:**

La mayoría de los usuarios de Atención Primaria de São Leopoldo presentaban un riesgo cardiovascular entre bajo y moderado. La prescripción de estatinas se ajustaba a las directrices clínicas al tener en cuenta el riesgo en la toma de decisiones, pero alcanzó menos de un tercio de la muestra general.

**Ethical aspects**

This research respected ethical principles, having obtained the following approval data:

Research Ethics Committee: Universidade do Vale do Rio dos Sinos

Opinion number: 4701266

Approval date: 10/5/2021

Certificate of Submission for Ethical Appraisal: 45251821.6.0000.5344

Informed Consent Form: Data Transfer Agreement, since data from Information Systems was accessed.

## Introduction

Noncommunicable diseases are responsible for three-quarters of all deaths worldwide (1). Cardiovascular diseases account for nearly half of these deaths and have been the leading cause of death in Brazil since the 1990s (2-3).

Several factors influence cardiovascular diseases, including race/skin color and sex; however, modifiable risk factors, such as hypertension, known diabetes, obesity, high cholesterol, unhealthy diet, insufficient physical activity, and active smoking status, are the main contributors to global mortality and represent a significant economic burden for health systems (4-6). Although a large proportion of individuals at high cardiovascular risk still do not have these factors adequately controlled, cardiovascular prevention programs have already reduced mortality in several countries (7-8).

In 2016, the World Health Organization launched the Global HEARTS Initiative (Healthy lifestyle counseling, Evidence-based treatment protocols, Access to essential medicines and technology, Risk-based cardiovascular disease management, Team-based care, and Systems for monitoring), a structured approach for the prevention and management of cardiovascular diseases in Primary Health Care. Its name is an acronym representing its six strategic pillars: healthy habits, evidence, access, risk, teamwork, and systems for monitoring (9).

Cardiovascular risk can be calculated using various tools, and the HEARTS risk calculator—which Brazil adopted in 2021—appears to reflect the national context better (10). This tool uses population-based data for Brazil and parameters defined by the Global Burden of Disease study. It includes the following variables: sex, known diabetes, active smoking status, age, systolic blood pressure, total cholesterol, and body mass index (BMI) (9). Although local guidelines and professional societies do not yet recommend it, the Cardiovascular Health Strategy in Primary Health Care considers the use of this tool timely and easily applicable, particularly due to its use of BMI in the calculation (11).

Risk stratification of Primary Health Care users enables the screening and monitoring of cardiovascular risk in a larger number of individuals, in addition to supporting prevention efforts, risk-based treatment adjustment, and better resource management (12). One of the key actions for the primary prevention of cardiovascular diseases is reducing serum levels of low-density lipoprotein (LDL) cholesterol through the use of medications such as drugs belonging to the statin class (13-18).

This study aimed to present cardiovascular risk strata based on the Global HEARTS Initiative calculator and to analyze statin prescription among Primary Health Care users in São Leopoldo, Rio Grande do Sul.

## Methods

### Study design

This was a cross-sectional observational study.

### Setting

The population comprises users who received medical treatment at Primary Health Care units in São Leopoldo between 2018 and 2021, as documented in the Cardiovascular Risk Operational Report. This report includes users with risk factors for cardiovascular diseases recorded in the Brazilian Citizens’ Electronic Health Record or the municipal health department’s Simplified Data Collection system. Data were collected between 2022 and 2023.

### Participants

Participants were selected based on records from the Cardiovascular Risk Operational Report, which employed a convenience sampling approach. The sample included all medical records of users aged 18 years or older for whom the variables required to determine cardiovascular risk were available (known diabetes, sex, active smoking status, age, systolic blood pressure, and either total cholesterol or BMI). Regardless of the year of registration, the most recent data were used. Records of users with inactive registrations were excluded from the study.

### Variables

The following data were collected: age (in years); sex (male, female); race/skin color (White, Black, Brown, Asian, Indigenous); education level (0-8, 9-11, ≥12 years); history of morbidities (hypertension, known diabetes, obesity, active smoking status); biochemical variables (total cholesterol; low-density lipoprotein cholesterol; high-density lipoprotein cholesterol; glucose, casual capillary blood glucose; glycated hemoglobin); blood pressure variables (systolic and diastolic blood pressure); anthropometric measurements (weight, height, BMI); and record of statin prescription (yes, no).

The presence of a clinical diagnosis recorded in the Brazilian Citizen’s Electronic Health Record or a BMI≥30 kg/m^2^ was considered history of obesity. The level of education reported in the Individual Registration Form was classified into years of study (0-8; 9-11; ≥12 years). Missing information was classified as “No information.” The following education classifications were recoded as 0–8 years of study “Literacy class – CA”, “Elementary school (1st to 4th grade)”, “Middle school (5th to 8th grade)”, “Complete primary school”, “Special elementary school”, “Youth and Adult Education - Elementary school (1st to 4th grade)”, “Youth and Adult Education - Middle School (5th to 8th grade)”, “Adult literacy (e.g. Brazilian Movement for Literacy - MOBRAL, etc.)” and “None.” 

### Data sources and measurement

Data were collected from the Brazilian Citizens’ Electronic Health Record, the Brazilian Simplified Data Collection system, and the Cardiovascular Risk Operational Report.

Total cholesterol values were converted from mg/dL to mmol/L by multiplying by 0.02586, as recommended in the WHO technical package for cardiovascular disease management in primary health care (9). Participants were classified as above or below the therapeutic target for low-density lipoprotein cholesterol of 100 mg/dL (14).

Based on the information obtained, the cardiovascular risk of patients was calculated according to the World Health Organization charts for the HEARTS initiative calculator (9). The calculation was performed in three stages, as follows.

1. Patients were categorized by known diabetes status, sex, active smoking status, age group, systolic blood pressure, and total cholesterol or BMI (if total cholesterol was unavailable).
2. The corresponding risk percentage was then determined by intersecting these variables in the tables.
3. Considering the percentage, patients were stratified as follows: low 10-year cardiovascular event risk if <5.0%; intermediate risk, 5.0–10.0%; high risk, 20.0–30.0%; and very high risk if ≥30.0%.

Since the lowest age range on the calculator is 40-44, participants aged 18-39 were included in the said range. It is worth noting that 64.9% of individuals aged 18-44 years were actually between 18 and 39 years old.

### Bias 

To reduce data collection bias, an integrated database was developed, and data collectors were trained in accessing, searching, and correctly coding the data. The following strategies were adopted to minimize potential data biases: the database was reviewed to ensure accurate coding; patterns were identified to explain the reasons for missing data; and associations between missing and observed data were examined. To handle missing data, absent information on active smoking status, known diabetes, and statin prescription was considered as “no history”.

### Study Size

The sample size was determined based on the availability of the data required for the study. Since the data had already been collected, sample size calculation was performed retrospectively. 

The analysis included 4,063 medical records. Of these, 54.1% contained all the variables necessary to calculate cardiovascular risk, corresponding to 2,199 patients, with a statin prescription frequency of 29.5%. With a 95% confidence level (Z=1.96), the margins of error were 1.5% for cardiovascular risk and 1.9% for statin prescription.

### Quantitative variables

The following units of measurement were used to describe cardiovascular risk factors: age in years; total cholesterol in millimoles per liter (mmol/L) for characterization and in milligrams per deciliter (mg/dL) for the remaining results; low-density lipoprotein (LDL) cholesterol, high-density lipoprotein (HDL) cholesterol, glucose, and casual capillary blood glucose in mg/dL; glycated hemoglobin in percentage (%); systolic and diastolic blood pressure in millimeters of mercury (mmHg); height in meters (m); weight in kilograms (kg); and BMI in kilograms per square meter (kg/m^2^).

### Statistical methods

Continuous data were presented as median (Med) and quartiles (Q1; Q3), and categorical data as absolute and relative frequencies. Frequencies and 95% confidence intervals (95%CI) were calculated for cardiovascular risk, statin prescription, and sociodemographic characteristics.

Normality of data distribution was tested using the Kolmogorov-Smirnov and Shapiro-Wilk tests. Due to the nonparametric distribution, the Mann-Whitney U test was used to compare age, total cholesterol, LDL cholesterol, HDL cholesterol, glucose, casual capillary blood glucose, glycated hemoglobin, systolic blood pressure, diastolic blood pressure, height, weight, BMI, and risk percentage between male and female users overall and stratified by cardiovascular risk. The significance level for this test is presented in the Results section, and quantitative variable values are shown in Tables 2 and 3.

Pearson’s chi-square test [x^2^(degrees of freedom)=test value; significance level] or Fisher’s exact test, when appropriate, was used to assess associations between cardiovascular risk and education level or morbidity history; between sex and cardiovascular risk or morbidity history; and between statin prescription and cardiovascular risk, sex, and low-density lipoprotein cholesterol therapeutic target.

Analyses were performed using IBM Statistical Package for the Social Sciences (version 25.0), with a significance level of 5.0% (p-value≤0.050).

## Results 

Cardiovascular risk was calculated for 2,199 users. Of these, 71.1% were female, with a median age of 56 (47; 65) years. Of the medical records that contained other characterizing information, most users were white (83.4%), had attended a maximum of 8 years of schooling (61.8%), and had a history of hypertension (81.9%).

The overall sample had a median total cholesterol level between 4.0 and 4.9 mmol/L (154.7–189.5 mg/dL), systolic blood pressure between 120 and 139 mmHg, and a BMI between 30 and 35 kg/m^2^. Most users had low cardiovascular risk (41.4%; 95%CI 9,3; 43,5), followed by moderate risk (38.0%; 95%CI 36,0; 40,1), high risk (19.6%; 95%CI 18,0; 21,3), and very high risk (1.0%; 95%CI 0,6; 1,4) (Table 1).

**Table 1:** Absolute frequencies (n), relative frequencies (%), and 95% confidence interval (95%CI) of cardiovascular risk by risk factor. São Leopoldo, 2018-2021 (n=2,199)

	Cardiovascular risk								
	Low		Moderate		High		Very high		
	n (%)	(95%CI)	n (%)	(95%CI)	n (%)	(95%CI)	n (%)	(95%CI)	p-value
**Age group** (years)									-
18-39	271 (29.8)	(26.9; 32.8)	22 (2.6)	(1.7; 3.9)	1 (0.2)	(0.0; 1.1)	-	-	
40-44	143 (15.7)	(13.5; 18.2)	16 (1.9)	(1.1; 3.0)	-	-	-	-	
45-49	162 (17.8)	(15.4; 20.4)	51 (6.1)	(4.6; 7.9)	7 (1.6)	(0.7; 3.2)	-	-	
50-54	171 (18.8)	(16.4; 21.4)	102 (12.2)	(10.1; 14.5)	7 (1.6)	(0.7; 3.2)	-	-	
55-59	136 (14.9)	(12.7; 17.4)	174 (20.8)	(18.2; 23.7)	34 (7.9)	(5.6; 10.7)	1 (4.8)	(0.5; 20.2)	
60-64	27 (3.0)	(2.0; 4.2)	227 (27.2)	(24.2; 30.2)	78 (18.1)	(14.6; 21.9)	3 (14.3)	(4.2; 33.4)	
65-69	-	-	153 (18.3)	(15.8; 21.0)	111 (25.7)	(21.7; 30.0)	4 (19.0)	(6.8; 39.2)	
≥70	-	-	91 (10.9)	(8.9; 13.1)	194 (44.9)	(40.3; 49.6)	13 (61.9)	(40.7; 80.1)	
Sex									<0.001
Female	763 (83.8)	(81.3; 86.1)	589 (70.5)	(67.3; 73.5)	210 (48.6)	(43.9; 53.3)	2 (9.5)	(2.0; 27.2)	
Male	147 (16.2)	(13.9; 18.7)	247 (29.5)	(26.5; 32.7)	222 (51.4)	(46.7; 56.1)	19 (90.5)	(72.8; 98.0)	
**Race/skin color**									-
White	751 (82.5)	(80.0; 84.9)	706 (84.5)	(81.9; 86.8)	359 (83.1)	(79.4; 86.4)	19 (90.5)	(72.8; 98.0)	
Black	58 (6.4)	(4.9; 8.1)	53 (6.3)	(4.8; 8.1)	38 (8.8)	(6.4; 11.7)	2 (9.5)	(2.0; 27.2)	
Brown	92 (10.1)	(8.3; 12.2)	70 (8.4)	(6.6; 10.4)	30 (6.9)	(4.8; 9.6)	-	-	
Asian	5 (0.6)	(0.2; 1.2)	4 (0.5)	(0.2; 1.1)	1 (0.2)	(0.0; 1.1)	-	-	
Indigenous	-	-	-	-	-	-	-	-	
No information	4 (0.4)	(0.2; 1.0)	3 (0.4)	(0.1; 1.0)	4 (0.9)	(0.3; 2.2)	-	-	
**Education level** (years)									<0.001
0-8	517 (56.8)	(53.6; 60.0)	548 (65.6)	(62.3; 68.7)	279 (64.6)	(60.0; 69.0)	14 (66.7)	(45.4; 83.7)	
9-11	71 (7.8)	(6.2; 9.7)	26 (3.1)	(2.1; 4.5)	11 (2.6)	(1.4; 4.4)	-	-	
≥12	10 (1.1)	(0.6; 1.9)	3 (0.4)	(0.1; 1.0)	1 (0.2)	(0.0; 1.1)	-	-	
No information	312 (34.3)	(31.3; 37.4)	259 (31.0)	(27.9; 34.2)	141 (32.6)	(28.3; 37.2)	7 (33.3)	(16.3; 54.6)	
**Known hypertension**									<0.001
Yes	680 (74.7)	(71.8; 77.5)	713 (85.3)	(82.8; 87.6)	390 (90.3)	(87.2; 92.8)	18 (85.7)	(66.6; 95.8)	
No	184 (20.2)	(17.7; 22.9)	102 (12.2)	(10.1; 14.6)	33 (7.6)	(5.4; 10.4)	3 (14.3)	(4.2; 33.4)	
No information	46 (5.1)	(3.8; 6.6)	21 (2.5)	(1.6; 3.7)	9 (2.1)	(1.0; 3.8)	-	-	
**Known diabetes**									<0.001
Yes	226 (24.8)	(22.1; 27.7)	351 (42.0)	(38.7; 45.4)	233 (53.9)	(49.2; 58.6)	15 (71.4)	(50.3; 87.1)	
No	684 (75.2)	(72.3; 77.9)	485 (58.0)	(54.6; 61.3)	199 (46.1)	(41.4; 50.8)	6 (28.6)	(12.9; 49.7)	
Obesity									<0.001
Yes	496 (54.5)	(51.3; 57.7)	378 (45.2)	(41.9; 48.6)	181 (41.9)	(37.3; 46.6)	11 (52.4)	(31.9; 72.3)	
No	354 (38.9)	(35.8; 42.1)	378 (45.2)	(41.9; 48.6)	197 (45.6)	(40.9; 50.3)	8 (38.1)	(19.9; 59.3)	
No information	60 (6.6)	(5.1; 8.3)	80 (9.6)	(7.7; 11.7)	54 (12.5)	(9.6; 15.9)	2 (9.5)	(2.0; 27.2)	
**Active smoking status**									<0.001
Yes	91 (10.0)	(8.2; 12.1)	145 (17.3)	(14.9; 20.0)	96 (22.2)	(18.5; 26.3)	10 (47.6)	(27.7; 68.1)	
No	819 (90.0)	(87.9; 91.8)	691 (82.7)	(80.0; 85.1)	336 (77.8)	(73.7; 81.5)	11 (52.4)	(31.9; 72.3)	
**Total cholesterol range** (mmol/L)									-
<4.0	76 (8.4)	(6.7; 10.3)	55 (6.6)	(5.1; 8.4)	46 (10.7)	(8.0; 13.8)	2 (9.5)	(2.0; 27.2)	
4.0-4.9	121 (13.3)	(11.2; 15.6)	123 (14.7)	(12.4; 17.2)	77 (17.8)	(14.4; 21.6)	6 (28.6)	(12.9; 49.7)	
5.0-5.9	85 (9.3)	(7.6; 11.4)	108 (12.9)	(10.8; 15.3)	59 (13.7)	(10.7; 17.1)	3 (14.3)	(4.2; 33.4)	
6.0-6.9	50 (5.5)	(4.2; 7.1)	62 (7.4)	(5.8; 9.3)	32 (7.4)	(5.2; 10.2)	-	-	
≥7.0	10 (1.1)	(0.6; 1.9)	22 (2.6)	(1.7; 3.9)	12 (2.8)	(1.5; 4.7)	2 (9.5)	(2.0; 27.2)	
No information	568 (62.4)	(59.2; 65.5)	466 (55.7)	(52.4; 59.1)	206 (47.7)	(43.0; 52.4)	8 (38.1)	(19.9; 59.3)	
**Systolic blood pressure** (mmHg)									-
<120	236 (25.9)	(23.2; 28.9)	103 (12.3)	(10.2; 14.7)	22 (5.1)	(3.3; 7.5)	-	-	
120-139	479 (52.6)	(49.4; 55.9)	412 (49.3)	(45.9; 52.7)	110 (25.5)	(21.5; 29.7)	-	-	
140-159	163 (17.9)	(15.5; 20.5)	254 (30.4)	(27.3; 33.6)	176 (40.7)	(36.2; 45.4)	3 (14.3)	(4.2; 33.4)	
160-179	29 (3.2)	(2.2; 4.5)	55 (6.6)	(5.0; 8.4)	91 (21.1)	(17.4; 25.1)	8 (38.1)	(19.9; 59.3)	
≥180	3 (0.3)	(0.1; 0.9)	12 (1.4)	(0.8; 2.4)	33 (7.6)	(5.4; 10.4)	10 (47.6)	(27.7; 68.1)	
**Body mass index range** (kg/m^2^)									-
<20	16 (1.8)	(1.0; 2.8)	17 (2.0)	(1.2; 3.2)	6 (1.4)	(0.6; 2.8)	-	-	
20-24	94 (10.3)	(8.5; 12.4)	105 (12.6)	(10.4; 14.9)	61 (14.1)	(11.1; 17.6)	1 (4.8)	(0.5; 20.2)	
25-29	242 (26.6)	(23.8; 29.5)	256 (30.6)	(27.6; 33.8)	129 (29.9)	(25.7; 34.3)	7 (33.3)	(16.3; 54.6)	
30-35	232 (25.5)	(22.7; 28.4)	235 (28.1)	(25.1; 31.2)	99 (22.9)	(19.1; 27.1)	9 (42.9)	(23.7; 63.8)	
≥35	256 (0.3)	(0.3; 0.3)	134 (0.2)	(0.1; 0.2)	78 (0.2)	(0.1; 0.2)	2 (0.1)	(0.0; 0.3)	
No information	70 (7.7)	(6.1; 9.6)	89 (10.6)	(8.7; 12.9)	59 (13.7)	(10.7; 17.1)	2 (9.5)	(2.0; 27.2)	
Total	910 (41.4)	(39.3; 43.5)	836 (38.0)	(36.0; 40.1)	432 (19.6)	(18.0; 21.3)	21 (1.0)	(0.6; 1.4)	

Cardiovascular risk was significantly associated with a history of obesity [x^2^(3)=16.63; p-value 0.001], being more frequent among users with low risk (54.5%). The history of diabetes was significantly associated with risk and was more frequent with higher risk [x^2^(3)=129.53; p-value<0.001], with a frequency of 71.4% in patients with very high risk. The same pattern was observed for active smoking status [x^2^(3)=54.47; p-value<0.001], with a prevalence of 47.6% in very high-risk patients, and history of hypertension, which increased up to the high-risk group (90.3%) [x^2^(3)=47.52; p-value<0.001], according to Fisher’s exact test.

Education level was also significantly associated with cardiovascular risk [x^2^(6)=38.08; p-value<0.001], as determined by Fisher’s exact test. Among users with no or low education level (0–8 years of schooling), 61.9% had moderate to very high cardiovascular risk. Users with 9–11 and ≥12 years of education mostly had low risk (65.7% and 71.4%, respectively).

The sex variable showed a significant association with cardiovascular risk [x^2^(3)=217.30; p-value<0.001] (Table 2). Male users accounted for the majority of the very high (90.5%) and high-risk (51.4%) groups. Among female participants, 48.8% had a low risk, 37.7% a moderate risk, 13.4% a high risk, and 0.1% a very high risk. Among male users, 23.1% had a low risk, 38.9% a moderate risk, 35.0% a high risk, and 3.0% a very high risk.

**Table 2:** Absolute frequencies (n), relative frequencies (%), and 95% confidence interval (95%CI) of sex by cardiovascular risk and history of morbidities. São Leopoldo, 2018-2021 (n=2,199)

	Female		Male		
	n (%)	(95%CI)	n (%)	(95%CI)	p-value
**Cardiovascular risk**					<0.001
Low	763 (48.8)	(46.3; 51.3)	147 (23.1)	(20.0; 26.5)	
Moderate	589 (37.7)	(35.3; 40.1)	247 (38.9)	(35.2; 42.7)	
High	210 (13.4)	(11.8; 15.2)	222 (35.0)	(31.3; 38.7)	
Very high	2 (0.1)	(0.0; 0.4)	19 (3.0)	(1.9; 4.5)	
**History of hypertension**					>0.050
Yes	1,271 (81.3)	(79.3; 83.1)	530 (83.5)	(80.4; 86.2)	
No	231 (14.8)	(13.1; 16.6)	91 (14.3)	(11.8; 17.2)	
No information	62 (4.0)	(3.1; 5.0)	14 (2.2)	(1.3; 3.6)	
**History of diabetes**					0.021
Yes	563 (36.0)	(33.6; 38.4)	262 (41.3)	(37.5; 45.1)	
No	1,001 (64.0)	(61.6; 66.4)	373 (58.7)	(54.9; 62.5)	
**History of obesity**					<0.001
Yes	828 (52.9)	(50.5; 55.4)	238 (37.5)	(33.8; 41.3)	
No	617 (39.5)	(37.0; 41.9)	320 (50.4)	(46.5; 54.3)	
No information	119 (7.6)	(6.4; 9.0)	77 (12.1)	(9.8; 14.8)	
**Active smoking status**					>0.050
Yes	242 (15.5)	(13.7; 17.3)	100 (15.7)	(13.1; 18.7)	
No	1,322 (84.5)	(82.7; 86.3)	535 (84.3)	(81.3; 86.9)	
Total	1,564 (100.0)	-	635 (100.0)	-	

Sex was also associated with a history of diabetes [x^2^(1)=5.33; p-value 0.021], being more frequent in male patients (41.3%), and with a history of obesity [x^2^(1)=34.69; p-value<0.001], which was more common among female patients (52.9%). No significant associations were found between sex and history of hypertension or active smoking status (p-value>0.050).

The overall values for total cholesterol, low-density lipoprotein cholesterol, high-density lipoprotein cholesterol, and BMI were higher among female patients. Male ones had higher values for age, glucose, systolic and diastolic blood pressure, height, weight, and risk percentage (Table 3).

**Table 3:** Median (Med) and quartiles (Q1; Q3) of risk factors and risk percentage between sexes. São Leopoldo, 2018-2021 (n=2,199)

	Female		Male		
	Med	(Q1; Q3)	Med	(Q1; Q3)	p-value
**Age** (years)	55	(46; 64)	59	(50; 65)	<0.001
**Total cholesterol** (mg/dL)	193	(167; 228)	181	(153; 209)	<0.001
**LDL-ca** (mg/dL)	107	(84; 132)	102	(78; 126)	0.030
**HDL-cb** (mg/dL)	52	(44; 62)	44	(37; 51)	<0.001
**Glucose** (mg/dL)	101	(89; 133)	106	(93; 136)	0.009
**Casual capillary blood glucose** (mg/dL)	157	(117; 225)	156	(119; 242)	0.983
**Glycated hemoglobin** (%)	6.5	(5.8; 10.5)	7.1	(5.9; 10.0)	0.330
**Systolic blood pressure** (mmHg)	130	(120; 140)	130	(120; 140)	<0.001
**Diastolic blood pressure** (mmHg)	80	(70; 90)	80	(70; 90)	0.014
**Height** (m)	1.56	(1.51; 1.6)	1.68	(1.63; 1.73)	<0.001
**Weight** (kg)	74.5	(64.5; 86)	80.4	(72; 92.5)	<0.001
**Body mass index** (kg/m^2^)	30.9	(26.7; 35.4)	29.1	(26.4; 32.7)	<0.001
**Risk percentage** (%)	5.0	(3.0; 8.0)	8.0	(5.0; 11.0)	<0.001

^a^LDL-c: low-density lipoprotein cholesterol, ^b^HDL-c: high-density lipoprotein cholesterol.

After risk stratification (Table 4), sex differences were observed. For low-risk patients, the variables high-density lipoprotein cholesterol, height, BMI, and risk percentage showed significant differences (p-value <0.001), as did age (p-value 0.003). In the moderate-risk group, significant differences were observed for age, high-density lipoprotein cholesterol, height, weight (p-value<0.001), and total cholesterol (p-value 0.001). In the high-risk group, significant differences were found for age, total cholesterol, high-density lipoprotein cholesterol, height, weight (p-value<0.001), risk percentage (p-value 0.004), low-density lipoprotein cholesterol (p-value 0.025), systolic blood pressure (p-value 0.010), and BMI (p-value 0.017). It was not possible to analyze differences in the very high-risk group due to the small number of female participants (n=2).

**Table 4:** Median (Med) and quartiles (Q1; Q3) of risk factors and risk percentage by cardiovascular risk and sex. São Leopoldo, 2018-2021 (n=2,199)

	Cardiovascular risk														
	Low					Moderate					High				
	Female		Male			Female		Male			Female		Male		
	Med	(Q1; Q3)	Med	(Q1; Q3)	p-value	Med	(Q1; Q3)	Med	(Q1; Q3)	p-value	Med	(Q1; Q3)	Med	(Q1; Q3)	p-value
**Age** (years)	46	(38; 53)	44	(35; 50)	0.003	62	(56; 67)	58	(53; 62)	<0.001	71	(66; 75)	66	(61; 72)	<0.001
**Total cholesterol** (mg/dL)	188	(160; 218)	177	(148; 214)	0.099	201	(174; 233)	185	(163; 209)	0.001	196	(173; 230)	180	(148; 204)	<0.001
**LDL-ca** (mg/dL)	105	(79; 130)	97	(70.5; 127.5)	0.529	110	(86; 136)	105	(90.5; 123.5)	0.398	110	(87; 134)	99	(69; 130)	0.025
**HDL-cb** (mg/dL)	50	(43; 59)	43	(36; 50)	<0.001	53	(46; 62)	44	(36; 52)	<0.001	56	(46; 65)	44	(38; 51)	<0.001
**Glucose** (mg/dL)	96	(86; 111)	96	(88; 102)	0.787	105	(91; 150)	109	(94; 146)	0.529	112	(95; 155)	113	(101; 161)	0.346
**Casual capillary blood glucose** (mg/dL)	139	(110; 202)	142	(103; 281)	0.631	166	(122; 247)	157	(120; 253)	0.480	170	(125; 212)	153	(121; 213)	0.662
**Glycated hemoglobin** (%)	6.0	(5.7; 11.4)	6.0	(5.2; 56.05)	0.809	7.0	(5.9; 11.0)	7.0	(5.85; 9.32)	0.446	7.0	(5.85; 9.6)	7.0	(6.2; 10.0)	0.146
**Systolic blood pressure** (mmHg)	120	(111; 130)	120	(110; 130)	0.548	130	(120; 140)	130	(120; 140)	0.566	140	(140; 160)	140	(130; 150)	0.010
**Diastolic blood pressure** (mmHg)	80	(70; 80)	80	(70; 80)	0.271	80	(70; 90)	80	(70; 90)	0.208	80	(80; 90)	80	(80; 90)	0.133
**Height** (m)	1.57	(1.53; 1.62)	1.69	(1.65; 1.75)	<0.001	1.54	(1.5; 1.59)	1.67	(1.63; 1.72)	<0.001	1.52	(1.48; 1.57)	1.67	(1.62; 1.72)	<0.001
**Weight** (kg)	79	(68.5; 92.5)	80	(70.4; 92)	0.143	71	(62.2; 82)	81	(74; 93)	<0.001	70	(61; 82)	79	(70; 91)	<0.001
**Body mass index** (kg/m^2^)	31.9	(27.5; 36.7)	29.0	(25.9; 32.1)	<0.001	30.1	(26.1; 33.7)	29.2	(26.8; 33.1)	0.246	30.7	(26.5; 35.2)	29.0	(25.9; 32.6)	0.017
**Risk percentage** (%)	3.0	(2.0; 4.0)	3.0	(2.0; 4.0)	<0.001	7.0	(5.0; 8.0)	7.0	(6.0; 8.0)	0.113	11.0	(10.0; 13.0)	12.0	(11.0; 14.0)	0.004

^a^LDL-c: low-density lipoprotein cholesterol, ^b^HDL-c: high-density lipoprotein cholesterol.

Statin prescription was recorded in 29.5% (95%CI 27.6; 31.4) of the medical records. A total of 53.3% (95%CI 49,9; 56,8) of users were above the LDL cholesterol therapeutic target; of these, 52.1% (95%CI 47.4; 56.8) had no statin prescription recorded.

There was a significant association [x^2^(3)=83.19; p-value<0.001] between statin prescription and cardiovascular risk, with a higher frequency among high-risk patients (41.2%) (Table 5). A significant association was also found between statin prescription and low-density lipoprotein cholesterol target [x^2^(1)=7.82; p-value 0.005], with a higher prescription rate among patients below the therapeutic target (51.6%). More than half (51.5%; 95%CI 46.4; 56.7) of patients above the low-density lipoprotein cholesterol target and with moderate to very high risk had no statin prescription recorded.

**Table 5:** Absolute frequencies (n) and relative frequencies (%) and 95% confidence intervals (95%CI) of statin prescriptions by sex, therapeutic target for low-density lipoprotein cholesterol, and cardiovascular risk. São Leopoldo, 2018-2021 (n=2,199)

	Prescription os statins					
	Yes		No		Total	
	n (%)	(95%CI)	n (%)	(95%CI)	n (%)	p-value
Sex						0.416
Female	453 (29.0)	(26.8; 31.2)	1,111 (71.0)	(68.8; 73.2)	1,564 (100.0)	
Male	195 (30.7)	(27.2; 34.4)	440 (69.3)	(65.6; 72.8)	635 (100.0)	
**LDL-c therapeutic target** ^a^						0.224
Below	165 (43.7)	(38.7; 48.7)	213 (56.3)	(51.3; 61.3)	378 (100.0)	
Above	207 (47.9)	(43.2; 52.6)	225 (52.1)	(47.4; 56.8)	432 (100.0)	
**Cardiovascular risk**						<0.001
Low	176 (19.3)	(16.9; 22.0)	734 (80.7)	(78.0; 83.1)	910 (100.0)	
Moderate	287 (34.3)	(31.2; 37.6)	549 (65.7)	(62.4; 68.8)	836 (100.0)	
High	178 (41.2)	(36.6; 45.9)	254 (58.8)	(54.1; 63.4)	432 (100.0)	
Very high	7 (33.3)	(16.3; 54.6)	14 (66.7)	(45.4; 83.7)	21 (100.0)	

^a^LDL-c: low-density lipoprotein cholesterol.

No association was found between statin prescription and sex in the overall sample (p-value>0.050). However, after stratifying participants above the low-density lipoprotein cholesterol target, a significant association with sex was found [x^2^(1)=4.19; p-value 0.041], with a higher frequency among male patients (59.8%).

## Discussion 

Only 54.1% of patients had sufficient information to allow for cardiovascular risk classification, despite the Global HEARTS initiative calculator permitting the use of BMI instead of total cholesterol. This situation limits the robustness of the analyses, may compromise the extrapolation of the cardiovascular risk profile found, and highlights the need to improve records, as this data supports actions to reduce the rates of complications, hospitalizations, and morbidity and mortality related to cardiovascular diseases (11).

Even though the HEARTS initiative calculator is recommended for individuals aged 40 years or older, assessing cardiovascular risk among users aged 18-39 years may offer benefits that outweigh the potential classification bias. This situation expands the analysis of the sample profile and the epidemiological landscape, aligned with the strategic action plan for tackling noncommunicable chronic diseases and conditions in Brazil, 2021-2030 (12).

Through this study, it was possible to identify that most participants presented low risk, which may be related to the predominance of females in the sample profile. Although differences in calculation methods may hinder direct comparison with previous studies, the cardiovascular risk observed in this study resembles the 10-year estimate for the Brazilian population, where males and individuals with no or low educational levels presented a higher risk (19). This increased risk may be associated with the biological predispositions of the male sex, lifestyle habits, and socioeconomic disadvantages linked to having no or low educational levels, such as limited access to services and prevention and health promotion practices (19-21).

Whereas the sample included users with some cardiovascular risk factors, a higher frequency of hypertension, diabetes, and active smoking status was observed with increasing risk levels. Female patients showed a higher frequency of obesity history and higher BMI values, which is consistent with the national and regional scenario (22-23), even though this sample presented greater differences between sexes, which may be related to data incompleteness, especially among male users.

The association between a history of obesity and cardiovascular risk was atypical, occurring more frequently in the low-risk group. This may be due to sample selection, as the prevalence of obesity was higher among females, who comprised the majority of the low-risk stratum.

Diabetes history was more frequent among males, contrasting with the prevalence found at the regional and national levels (22,24). Blood pressure values were also higher among male patients, even though there was no difference in the frequency of hypertension history frequency between the sexes. It was also possible to identify the lipid profile of the patients treated in the municipality’s primary care services. The values for total cholesterol and its fractions in the general sample and by sex were similar to those found in the adult Brazilian population (25).

No association was found between sex and being above or below the low-density lipoprotein cholesterol target. Most of the sample presented low-density lipoprotein cholesterol values above the target. Females showed higher values of total cholesterol, high-density lipoprotein cholesterol, and low-density lipoprotein cholesterol. These differences may be related to climacteric and menopause, phases relevant to cardiovascular risk in women, which reinforces the need for screening and early interventions in this population (26-27).

The frequency of statin prescription was similar to that found in Brazilian Primary Health Care settings (31.1%) (23). In this study, the frequency of statin prescriptions was associated with the therapeutic target for low-density lipoprotein cholesterol. Prescription was more frequent among patients below the target, which may indicate low-density lipoprotein cholesterol reduction due to statin use. However, approximately half of the users above the low-density lipoprotein cholesterol target and with moderate to very high risk had no statin prescription recorded. This finding suggests possible barriers in care, such as difficulties in cardiovascular risk screening.

At the same time, there was an association between prescription and cardiovascular risk, with higher prescription frequency among those with higher risk. Since the purpose of statin use in primary prevention is to reduce cardiovascular risk, the prescription records appear consistent with the Global HEARTS initiative and with the Clinical Protocol and Therapeutic Guidelines for Dyslipidemia in effect during the study period (14-15).

As of 2025, a few lipid-lowering medications provided by the Brazilian National Health System may be added to benefit high-risk users who have not reached the low-density lipoprotein cholesterol target using statins alone (14). Simvastatin remains more commonly prescribed than atorvastatin, possibly due to the type of prescription required and the cost of the medications, which may limit access to optimized treatment (28).

Despite the free provision of medications for diabetes, hypertension, and dyslipidemia through the Brazilian National Health System, primary prevention policies remain insufficient to control cardiovascular risk factors (23).

The results of this study provide an overview of actions to reduce cardiovascular risk through the use of statins. The absence of analysis regarding which statin was prescribed, as well as data on diet and physical activity, may have limited further inferences. 

Early screening, pharmacological management, physical activity, healthy diet, and smoking cessation are fundamental to cardiovascular risk management. Even so, access barriers to health services hinder adherence and follow-up, which may lead to related complications (15,20-21). The low prevalence of ideal cardiovascular health in the Brazilian population highlights the disparity between actual care and ideal targets (29). This reveals a gap in the effectiveness of care delivery by the multidisciplinary health team compared to the expected ideal.

To improve comprehensive care for the prevention and control of cardiovascular diseases, municipal action plans must address health promotion and prevention, professional and managerial training, early diagnosis of morbidities, strengthening of management and infrastructure, and the promotion of research and innovation (11). These plans should prioritize continuing education initiatives to raise awareness among health professionals and, simultaneously, promote healthy eating, physical activity counseling, and tobacco cessation, along with intersectoral coordination for the social production of health through the promotion of sustainable development (30).

The results reinforce the need to plan additional cardiovascular risk screening strategies and preventive actions in São Leopoldo’s Primary Health Care system. Most users presented low to moderate cardiovascular risk, and statin prescription reached less than one-third of the overall sample, although it was aligned with clinical guidelines by considering risk in the decision-making process.
