# Supplementary material for: Cardiovascular risk and statin prescription in primary care: an observational study, São Leopoldo, 2018-2021
Source: Epidemiol Serv Saude. 2025 Oct 20;34:e20240432. doi: 10.1590/S2237-96222025v34e20240432.en (PMC12536959; doi:10.1590/S2237-96222025v34e20240432.en)
Supplement: Translation

# Translation: Risco cardiovascular e prescrição de estatinas na atenção primária: estudo observacional, São Leopoldo, 2018-2021

**DOI:** 10.1590/S2237-96222025v34e20240432.pt

**Authors:** Vania Celina Dezoti Micheletti, Roger dos Santos Rosa, Thiago Dipp, Luise Peter da Silva, Paula Dal Bó Campagnolo, Luís Richard Mercaus, Jéssica Rosiane de Brito, Maria Eduarda Moutinho Bonin

**Affiliations:** 1 Universidade do Vale do Rio do Sinos, Programa de Pós-Graduação em Saúde Coletiva, São Leopoldo, RS, Brasil; 2 Universidade Federal do Rio Grande do Sul, Faculdade de Medicina, Porto Alegre, RS, Brasil; 3 Universidade Federal do Rio Grande do Sul, Programa de Pós-Graduação em Saúde Coletiva, Porto Alegre, RS, Brasil; 4 Universidade do Vale do Rio do Sinos, Mestrado Profissional em Alimentos, Nutrição e Saúde, São Leopoldo, RS, Brasil; 5 Universidade do Vale do Rio do Sinos, Programa de Residência Multiprofissional em Atenção Básica, São Leopoldo, RS, Brasil; 6 Universidade Luterana do Brasil, Programa de Residência Multiprofissional de Saúde Comunitária, Canoas, RS, Brasil

**Keywords:** Prevenção Primária, Atenção Primária à Saúde, Doenças Cardiovasculares, Hipolipemiantes, Colesterol

## Abstract

**Objetivos:**

Apresentar os estratos de risco cardiovascular pela calculadora da iniciativa HEARTS e analisar a prescrição de estatinas para usuários da Atenção Primária à Saúde de São Leopoldo, Rio Grande do Sul.

**Métodos:**

Tratou-se de estudo observacional transversal que incluiu usuários com idade mínima de 18 anos presentes no Relatório Operacional de Risco Cardiovascular. Os dados categóricos foram apresentados em frequências absoluta e relativa e intervalos de confiança de 95% (IC95%). Para análise de associações entre as variáveis categóricas, foi utilizado o teste de qui-quadrado ou o teste exato de Fisher com significância estatística se p-valor≤0,050.

**Resultados:**

Foi possível realizar o cálculo do risco cardiovascular de 2.199 usuários. A maioria dos usuários apresentou risco baixo (41,4%; IC95% 39,3; 43,5) ou moderado (38,0%; IC95% 36,0; 40,1), enquanto 19,6% (IC95% 18,0; 21,3) apresentaram risco alto e 1,0% (IC95% 0,6; 1,4), risco muito alto. O registro da prescrição de estatinas constava em 29,5% (IC95% 27,6; 31,4) dos prontuários. Houve associação significativa (p-valor<0,001) entre o registro da prescrição de estatinas e o risco cardiovascular, com maior frequência em usuários com risco alto (41,2%).

**Conclusão:**

A maioria dos usuários da Atenção Primária de São Leopoldo apresentou risco cardiovascular entre baixo e moderado. A prescrição de estatinas estava alinhada com as diretrizes clínicas ao considerar o risco na tomada de decisão, mas alcançou menos de um terço da amostra geral.

**Aspectos éticos**

A presente pesquisa respeitou os princípios éticos, obtendo os seguintes dados de aprovação: :

Comitê de ética em pesquisa: Universidade do Vale do Rio dos Sinos

Número do parecer: 4.701.266

Data de aprovação: 10/5/2021

Certificado de apresentação de apreciação ética: 45251821.6.0000.5344

Registro do consentimento livre e esclarecido: Termo de Cedência de Dados, uma vez que foram acessados dados de Sistemas de Informação.

## Introdução 

As doenças crônicas não transmissíveis são responsáveis por três quartos das mortes em todo o mundo (1). As doenças cardiovasculares são a causa de quase metade dessas mortes e a principal causa de morte no Brasil desde a década de 1990 (2-3).

Diversos fatores influenciam as doenças cardiovasculares, como raça/cor da pele e sexo, mas os fatores de risco modificáveis, como hipertensão, diabetes, obesidade, colesterol elevado, dieta inadequada, sedentarismo e tabagismo, são os principais responsáveis pela mortalidade global e possuem grande carga econômica para o sistema de saúde (4-6). Embora grandes proporções de pessoas com alto risco cardiovascular ainda não apresentem controle adequado desses fatores, programas de prevenção cardiovascular já reduziram as mortes em vários países (7-8).

A Organização Mundial da Saúde desenvolveu em 2016 a iniciativa HEARTS (*Healthy-lifestyle counseling, Evidence-based treatment protocols, Access to essential medicines and technology, Risk-based cardiovascular disease management, Team-based care, and Systems for monitoring*), uma abordagem estruturada para a prevenção e o manejo de doenças cardiovasculares na Atenção Primária à Saúde. Seu nome, que não é traduzido, é um acrônimo dos seis pilares estratégicos: hábitos saudáveis; evidência; acesso; risco; trabalho em equipe; e sistemas para monitoramento (9).

O risco cardiovascular pode ser calculado por meio de diversas ferramentas, e a calculadora de risco cardiovascular da iniciativa HEARTS, a qual o Brasil aderiu em 2021, aparenta aproximar-se mais da realidade brasileira (10). Essa ferramenta da iniciativa HEARTS considera os dados de estimativa populacional brasileira e utiliza parâmetros definidos a partir do Global Burden of Disease. Ela inclui no cálculo as variáveis sexo, diabetes, tabagismo, idade, pressão arterial sistólica, colesterol total e índice de massa corporal (IMC) (9). Apesar de ainda não ser recomendada por diretrizes e sociedades locais, a Estratégia de Saúde Cardiovascular na Atenção Primária à Saúde entende a utilização dessa ferramenta como oportuna e de fácil aplicabilidade ao possibilitar o cálculo por meio do IMC (11).

A estratificação de risco dos usuários na Atenção Primária possibilita o rastreio e o monitoramento do risco cardiovascular do maior número de usuários, além da prevenção, adequação do tratamento baseado no risco e melhor gestão de recursos (12). Uma das ações de prevenção primária das doenças cardiovasculares é a redução dos níveis séricos de colesterol da lipoproteína de baixa densidade por meio de medicamentos, como os que integram a classe farmacológica das estatinas (13-18).

Este estudo teve como objetivo apresentar os estratos de risco cardiovascular pela calculadora da iniciativa HEARTS e analisar a prescrição de estatinas para usuários da Atenção Primária à Saúde de São Leopoldo, Rio Grande do Sul.

## Métodos 

### Desenho do estudo

Tratou-se de estudo do tipo observacional transversal.

### Contexto 

A população abrange usuários que receberam atendimento nas unidades de Atenção Primária à Saúde de São Leopoldo, no período 2018-2021, presentes no Relatório Operacional de Risco Cardiovascular. Este considera os usuários com fatores de risco para doenças cardiovasculares registrados no Prontuário Eletrônico do Cidadão ou na Coleta de Dados Simplificada da secretaria municipal de saúde. Os dados foram coletados entre 2022 e 2023.

### Participantes 

Os participantes foram selecionados com base nos registros do Relatório Operacional de Risco Cardiovascular, o que caracteriza a amostragem por conveniência. A amostra incluiu todos os prontuários de usuários com idade mínima de 18 anos que apresentassem as variáveis necessárias para a determinação do risco cardiovascular (diabetes, sexo, tabagismo, idade, pressão arterial sistólica e colesterol total ou IMC). Independentemente do ano do registro, foram utilizados os dados mais recentes. Foram excluídos do estudo prontuários de usuários com o cadastro inativo.

### Variáveis 

Foram coletados dados sobre: idade (em anos); sexo (masculino, feminino); raça/cor da pele (branca, preta, parda, amarela, indígena); escolaridade (0-8, 9-11, ≥12 anos); histórico de morbidades (hipertensão, diabetes, obesidade, tabagismo); variáveis bioquímicas (colesterol total; colesterol da lipoproteína de baixa densidade, colesterol da lipoproteína de alta densidade; glicose, glicemia capilar casual; hemoglobina glicada); variáveis pressóricas (pressão arterial sistólica, pressão arterial diastólica); medidas antropométricas (peso, altura, IMC); e registro da prescrição de estatinas (sim, não).

Como histórico de obesidade, foi considerada a presença do diagnóstico clínico no Prontuário Eletrônico do Cidadão ou IMC ≥30kg/m^2^. A escolaridade presente na Ficha de Cadastro Individual dos usuários foi classificada em anos de estudo (0-8; 9-11; ≥12 anos). Os casos omissos foram classificados como “Sem informação”, e os registros foram recodificados como de 0-8 anos de estudo em: “Classe de alfabetização – CA”, “Ensino fundamental 1ª a 4ª séries”, “Ensino fundamental 5ª a 8ª séries”, “Ensino fundamental completo”, “Ensino fundamental especial”, “Ensino fundamental EJA – séries iniciais (supletivo 1ª a 4ª)”, “Ensino fundamental EJA – séries finais (supletivo 5ª a 8ª)”, “Alfabetização para adultos (mobral etc.)” e “Nenhum”. 

### Fontes de dados e mensuração

Os dados foram coletados do Prontuário Eletrônico do Cidadão, da Coleta de Dados Simplificada e do Relatório Operacional de Risco Cardiovascular.

O valor de colesterol total foi convertido de mg/dL para mmol/L por meio da multiplicação por 0,02586, como recomendado no pacote de medidas técnicas para manejo da doença cardiovascular na Atenção Primária à Saúde da Organização Mundial de Saúde (9). Os participantes foram classificados acima ou abaixo do alvo terapêutico de colesterol da lipoproteína de baixa densidade de 100 mg/dL (14).

Por meio das informações obtidas, foi calculado o risco cardiovascular dos usuários, de acordo com as tabelas publicadas pela Organização Mundial da Saúde para a calculadora da iniciativa HEARTS (9). O cálculo ocorreu em três etapas, conforme a seguir.

1. Os usuários foram divididos pelo histórico de diabetes, sexo, histórico de tabagismo, faixa etária, pressão arterial sistólica e colesterol total ou IMC (se não houvesse registro do colesterol total).
2. Foi verificado o percentual de risco definido para a intersecção dessas variáveis nas tabelas.
3. A partir do percentual, os usuários foram estratificados em: baixo risco de evento cardiovascular em 10 anos se <5,0%; risco intermediário, entre 5,0 e 10,0%; risco alto, entre 20,0 e 30,0%; e risco muito alto caso apresentasse ≥30,0%.

Como a menor faixa etária da calculadora é 40-44 anos, os usuários com idade entre 18 e 39 anos foram considerados nessa faixa etária. Destacou-se que 64,9% dos indivíduos na faixa etária 18-44 anos possuíam entre 18 e 39 anos.

### Viés 

A fim de diminuir vieses de coleta, foi desenvolvido um banco de dados integrado, e os responsáveis pela coleta foram treinados para acesso, busca e correta codificação dos dados. Para minimizar possíveis vieses de dados, foram adotadas as seguintes estratégias: o banco de dados foi revisado para garantir que os dados foram codificados corretamente; foram buscados padrões que explicassem o motivo dos dados faltantes; e foram conferidas associações entre esses dados e outros observados. Para lidar com os dados faltantes, os casos omissos do histórico de tabagismo, diabetes e do registro da prescrição de estatinas foram considerados como sem histórico.

### Tamanho do estudo

A determinação do tamanho da amostra foi baseada na disponibilidade dos dados necessários para o estudo. Visto que os dados já haviam sido coletados, o cálculo do tamanho amostral foi realizado a posteriori. 

A análise considerou 4.063 prontuários. Desse total, 54,1% tinham todas as variáveis necessárias para calcular o risco cardiovascular. Esses prontuários correspondiam a 2.199 usuários, com a frequência de prescrição de estatinas de 29,5%. Com nível de confiança de 95% (Z=1,96), a margem de erro foi 1,5% para o risco cardiovascular e 1,9% para a prescrição de estatinas.

### Variáveis quantitativas

Para a descrição dos fatores de risco cardiovascular, foram utilizadas as seguintes unidades de medida: idade em anos; colesterol total em milimoles por litro (mmol/L) para caracterização e em miligramas por decilitro (mg/dL) nos demais resultados; colesterol da lipoproteína de baixa densidade, colesterol da lipoproteína de alta densidade, glicose e glicemia capilar casual em mg/dL; hemoglobina glicada em percentual (%); pressão arterial sistólica e pressão arterial diastólica em milímetros de mercúrio (mmHg); altura em metros (m); peso em quilogramas (kg); e IMC em quilogramas por metro quadrado (kg/m^2^).

### Métodos estatísticos

Os dados contínuos foram apresentados em mediana (Med) e quartis (Q1; Q3), e os dados categóricos, em frequências absoluta e relativa. As frequências e os intervalos de confiança de 95% (IC95%) foram calculados para o risco cardiovascular, assim como para a prescrição de estatinas e variáveis de características sociodemográficas.

A normalidade da distribuição dos dados foi analisada pelos testes de Kolmogorov-Smirnov e Shapiro-Wilk. Devido à distribuição não paramétrica dos dados, foi utilizado o teste U de Mann-Whitney para a comparação das variáveis idade, colesterol total, colesterol da lipoproteína de baixa densidade, colesterol da lipoproteína de alta densidade, glicose, glicemia capilar casual, hemoglobina glicada, pressão arterial sistólica, pressão arterial diastólica, altura, peso, IMC e percentual de risco entre o sexo masculino e feminino no geral e estratificado por risco cardiovascular. O valor do nível de significância desse teste foi apresentado na seção Resultados, e o valor das variáveis quantitativas, nas Tabelas 2 e 3.

Foi realizado o teste de qui-quadrado de Pearson [x^2^(grau de liberdade)=valor do teste; nível de significância] ou teste exato de Fisher, conforme apropriado, para analisar associações do risco cardiovascular com a escolaridade e o histórico de morbidades; do sexo com o risco cardiovascular e o histórico de morbidades; e da prescrição de estatinas com o risco cardiovascular, o sexo e o alvo terapêutico de colesterol da lipoproteína de baixa densidade.

As análises foram realizadas no software IBM Statistical Package for the Social Sciences (versão 25.0), e adotou-se como nível de significância estatística 5,0% (p-valor≤0,050).

## Resultados 

Foi possível realizar o cálculo do risco cardiovascular de 2.199 usuários. Destes, 71,1% eram do sexo feminino, com mediana de idade de 56 (47; 65) anos. Dos prontuários que possuíam outras informações de caracterização, a maioria dos usuários era da raça/cor da pele branca (83,4%), com até 8 anos de estudo (61,8%), e possuía histórico de hipertensão (81,9%).

A amostra geral apresentou a mediana do valor de colesterol total na faixa entre 4,0-4,9 mmol/L (154,7-189,5 mg/dL), de pressão arterial sistólica entre 120-139 mmHg e de IMC entre 30-35 kg/m^2^. A maior parte da amostra (41,4%; IC95% 39,3; 43,5) apresentou risco cardiovascular baixo; 38,0% (IC95% 36,0; 40,1), risco moderado; 19,6% (IC95% 18,0; 21,3), risco alto; e 1,0% (IC95% 0,6; 1,4), risco muito alto (Tabela 1).

**Tabela 1:** Frequências absoluta (n) e relativa (%) e intervalo de confiança de 95% (IC95%) do risco cardiovascular por fator de risco. São Leopoldo, 2018-2021 (n=2.199)

	Risco cardiovascular								
	Baixo		Moderado		Alto		Muito alto		
	n (%)	(IC95%)	n (%)	(IC95%)	n (%)	(IC95%)	n (%)	(IC95%)	p-valor
**Faixa etária** (anos)									-
18-39	271 (29,8)	(26,9; 32,8)	22 (2,6)	(1,7; 3,9)	1 (0,2)	(0,0; 1,1)	-	-	
40-44	143 (15,7)	(13,5; 18,2)	16 (1,9)	(1,1; 3,0)	-	-	-	-	
45-49	162 (17,8)	(15,4; 20,4)	51 (6,1)	(4,6; 7,9)	7 (1,6)	(0,7; 3,2)	-	-	
50-54	171 (18,8)	(16,4; 21,4)	102 (12,2)	(10,1; 14,5)	7 (1,6)	(0,7; 3,2)	-	-	
55-59	136 (14,9)	(12,7; 17,4)	174 (20,8)	(18,2; 23,7)	34 (7,9)	(5,6; 10,7)	1 (4,8)	(0,5; 20,2)	
60-64	27 (3,0)	(2,0; 4,2)	227 (27,2)	(24,2; 30,2)	78 (18,1)	(14,6; 21,9)	3 (14,3)	(4,2; 33,4)	
65-69	-	-	153 (18,3)	(15,8; 21,0)	111 (25,7)	(21,7; 30,0)	4 (19,0)	(6,8; 39,2)	
≥70	-	-	91 (10,9)	(8,9; 13,1)	194 (44,9)	(40,3; 49,6)	13 (61,9)	(40,7; 80,1)	
Sexo									<0,001
Feminino	763 (83,8)	(81,3; 86,1)	589 (70,5)	(67,3; 73,5)	210 (48,6)	(43,9; 53,3)	2 (9,5)	(2,0; 27,2)	
Masculino	147 (16,2)	(13,9; 18,7)	247 (29,5)	(26,5; 32,7)	222 (51,4)	(46,7; 56,1)	19 (90,5)	(72,8; 98,0)	
**Raça/cor da pele**									-
Branca	751 (82,5)	(80,0; 84,9)	706 (84,5)	(81,9; 86,8)	359 (83,1)	(79,4; 86,4)	19 (90,5)	(72,8; 98,0)	
Preta	58 (6,4)	(4,9; 8,1)	53 (6,3)	(4,8; 8,1)	38 (8,8)	(6,4; 11,7)	2 (9,5)	(2,0; 27,2)	
Parda	92 (10,1)	(8,3; 12,2)	70 (8,4)	(6,6; 10,4)	30 (6,9)	(4,8; 9,6)	-	-	
Amarela	5 (0,6)	(0,2; 1,2)	4 (0,5)	(0,2; 1,1)	1 (0,2)	(0,0; 1,1)	-	-	
Indígena	-	-	-	-	-	-	-	-	
Sem informação	4 (0,4)	(0,2; 1,0)	3 (0,4)	(0,1; 1,0)	4 (0,9)	(0,3; 2,2)	-	-	
**Escolaridade** (anos)									<0,001
0-8	517 (56,8)	(53,6; 60,0)	548 (65,6)	(62,3; 68,7)	279 (64,6)	(60,0; 69,0)	14 (66,7)	(45,4; 83,7)	
9-11	71 (7,8)	(6,2; 9,7)	26 (3,1)	(2,1; 4,5)	11 (2,6)	(1,4; 4,4)	-	-	
≥12	10 (1,1)	(0,6; 1,9)	3 (0,4)	(0,1; 1,0)	1 (0,2)	(0,0; 1,1)	-	-	
Sem informação	312 (34,3)	(31,3; 37,4)	259 (31,0)	(27,9; 34,2)	141 (32,6)	(28,3; 37,2)	7 (33,3)	(16,3; 54,6)	
**Histórico de hipertensão**									<0,001
Sim	680 (74,7)	(71,8; 77,5)	713 (85,3)	(82,8; 87,6)	390 (90,3)	(87,2; 92,8)	18 (85,7)	(66,6; 95,8)	
Não	184 (20,2)	(17,7; 22,9)	102 (12,2)	(10,1; 14,6)	33 (7,6)	(5,4; 10,4)	3 (14,3)	(4,2; 33,4)	
Sem informação	46 (5,1)	(3,8; 6,6)	21 (2,5)	(1,6; 3,7)	9 (2,1)	(1,0; 3,8)	-	-	
**Histórico de diabetes**									<0,001
Sim	226 (24,8)	(22,1; 27,7)	351 (42,0)	(38,7; 45,4)	233 (53,9)	(49,2; 58,6)	15 (71,4)	(50,3; 87,1)	
Não	684 (75,2)	(72,3; 77,9)	485 (58,0)	(54,6; 61,3)	199 (46,1)	(41,4; 50,8)	6 (28,6)	(12,9; 49,7)	
**Histórico de obesidade**									<0,001
Sim	496 (54,5)	(51,3; 57,7)	378 (45,2)	(41,9; 48,6)	181 (41,9)	(37,3; 46,6)	11 (52,4)	(31,9; 72,3)	
Não	354 (38,9)	(35,8; 42,1)	378 (45,2)	(41,9; 48,6)	197 (45,6)	(40,9; 50,3)	8 (38,1)	(19,9; 59,3)	
Sem informação	60 (6,6)	(5,1; 8,3)	80 (9,6)	(7,7; 11,7)	54 (12,5)	(9,6; 15,9)	2 (9,5)	(2,0; 27,2)	
**Histórico de tabagismo**									<0,001
Sim	91 (10,0)	(8,2; 12,1)	145 (17,3)	(14,9; 20,0)	96 (22,2)	(18,5; 26,3)	10 (47,6)	(27,7; 68,1)	
Não	819 (90,0)	(87,9; 91,8)	691 (82,7)	(80,0; 85,1)	336 (77,8)	(73,7; 81,5)	11 (52,4)	(31,9; 72,3)	
**Faixa de colesterol total** (mmol/L)									-
<4,0	76 (8,4)	(6,7; 10,3)	55 (6,6)	(5,1; 8,4)	46 (10,7)	(8,0; 13,8)	2 (9,5)	(2,0; 27,2)	
4,0-4,9	121 (13,3)	(11,2; 15,6)	123 (14,7)	(12,4; 17,2)	77 (17,8)	(14,4; 21,6)	6 (28,6)	(12,9; 49,7)	
5,0-5,9	85 (9,3)	(7,6; 11,4)	108 (12,9)	(10,8; 15,3)	59 (13,7)	(10,7; 17,1)	3 (14,3)	(4,2; 33,4)	
6,0-6,9	50 (5,5)	(4,2; 7,1)	62 (7,4)	(5,8; 9,3)	32 (7,4)	(5,2; 10,2)	-	-	
≥7,0	10 (1,1)	(0,6; 1,9)	22 (2,6)	(1,7; 3,9)	12 (2,8)	(1,5; 4,7)	2 (9,5)	(2,0; 27,2)	
Sem informação	568 (62,4)	(59,2; 65,5)	466 (55,7)	(52,4; 59,1)	206 (47,7)	(43,0; 52,4)	8 (38,1)	(19,9; 59,3)	
**Faixa de pressão arterial sistólica** (mmHg)									-
<120	236 (25,9)	(23,2; 28,9)	103 (12,3)	(10,2; 14,7)	22 (5,1)	(3,3; 7,5)	-	-	
120-139	479 (52,6)	(49,4; 55,9)	412 (49,3)	(45,9; 52,7)	110 (25,5)	(21,5; 29,7)	-	-	
140-159	163 (17,9)	(15,5; 20,5)	254 (30,4)	(27,3; 33,6)	176 (40,7)	(36,2; 45,4)	3 (14,3)	(4,2; 33,4)	
160-179	29 (3,2)	(2,2; 4,5)	55 (6,6)	(5,0; 8,4)	91 (21,1)	(17,4; 25,1)	8 (38,1)	(19,9; 59,3)	
≥180	3 (0,3)	(0,1; 0,9)	12 (1,4)	(0,8; 2,4)	33 (7,6)	(5,4; 10,4)	10 (47,6)	(27,7; 68,1)	
**Faixa de índice de massa corporal** (kg/m^2^)									-
<20	16 (1,8)	(1,0; 2,8)	17 (2,0)	(1,2; 3,2)	6 (1,4)	(0,6; 2,8)	-	-	
20-24	94 (10,3)	(8,5; 12,4)	105 (12,6)	(10,4; 14,9)	61 (14,1)	(11,1; 17,6)	1 (4,8)	(0,5; 20,2)	
25-29	242 (26,6)	(23,8; 29,5)	256 (30,6)	(27,6; 33,8)	129 (29,9)	(25,7; 34,3)	7 (33,3)	(16,3; 54,6)	
30-35	232 (25,5)	(22,7; 28,4)	235 (28,1)	(25,1; 31,2)	99 (22,9)	(19,1; 27,1)	9 (42,9)	(23,7; 63,8)	
≥35	256 (0,3)	(0,3; 0,3)	134 (0,2)	(0,1; 0,2)	78 (0,2)	(0,1; 0,2)	2 (0,1)	(0,0; 0,3)	
Sem informação	70 (7,7)	(6,1; 9,6)	89 (10,6)	(8,7; 12,9)	59 (13,7)	(10,7; 17,1)	2 (9,5)	(2,0; 27,2)	
Total	910 (41,4)	(39,3; 43,5)	836 (38,0)	(36,0; 40,1)	432 (19,6)	(18,0; 21,3)	21 (1,0)	(0,6; 1,4)	

Foi encontrada associação significativa do risco cardiovascular com o histórico de obesidade [x^2^(3)=16,63; p-valor 0,001], que foi mais frequente em usuários com risco baixo (54,5%). O histórico de diabetes apresentou associação significativa com o risco e foi mais frequente conforme maior risco [x^2^(3)=129,53; p-valor<0,001], com frequência de 71,4% em usuários com risco muito alto. Esse padrão também ocorreu com o histórico de tabagismo [x^2^(3)=54,47; p-valor<0,001], com a prevalência de 47,6% em usuários com risco muito alto, e com o histórico de hipertensão, que aumentou até o risco alto (90,3%) com associação significativa [x^2^(3)=47,52; p-valor<0,001] pelo teste exato de Fisher.

A escolaridade também estava significativamente associada com o risco cardiovascular [x^2^(6)=38,08; p-valor<0,001] pelo teste exato de Fisher. A maioria dos usuários com nenhuma ou baixa escolaridade (0-8 anos de estudo) apresentou risco cardiovascular entre moderado e muito alto (61,9%). Os usuários com 9 a 11 e igual ou superior a 12 anos de estudo apresentaram risco baixo (65,7% e 71,4%).

O sexo mostrou associação significativa com o risco cardiovascular [x^2^(3)=217,30; p-valor<0,001] (Tabela 2). Usuários do sexo masculino compuseram a maioria da amostra com risco muito alto (90,5%) e alto (51,4%). A maior parcela dos usuários do sexo feminino, equivalente a 48,8%, apresentou risco baixo; 37,7%, risco moderado; 13,4%, risco alto; e 0,1%, risco muito alto. Entre o sexo masculino, 23,1% dos usuários apresentaram risco baixo; 38,9%, risco moderado; 35,0%, risco alto; e 3,0%, risco muito alto.

**Tabela 2:** Frequências absoluta (n) e relativa (%) e intervalo de confiança de 95% (IC95%) do sexo por risco cardiovascular e histórico de morbidades. São Leopoldo, 2018-2021 (n=2.199)

	Sexo feminino		Sexo masculino		
	n (%)	(IC95%)	n (%)	(IC95%)	p-valor
**Risco cardiovascular**					<0,001
Baixo	763 (48,8)	(46,3; 51,3)	147 (23,1)	(20,0; 26,5)	
Moderado	589 (37,7)	(35,3; 40,1)	247 (38,9)	(35,2; 42,7)	
Alto	210 (13,4)	(11,8; 15,2)	222 (35,0)	(31,3; 38,7)	
Muito alto	2 (0,1)	(0,0; 0,4)	19 (3,0)	(1,9; 4,5)	
**Histórico de hipertensão**					>0,050
Sim	1.271 (81,3)	(79,3; 83,1)	530 (83,5)	(80,4; 86,2)	
Não	231 (14,8)	(13,1; 16,6)	91 (14,3)	(11,8; 17,2)	
Sem informação	62 (4,0)	(3,1; 5,0)	14 (2,2)	(1,3; 3,6)	
**Histórico de diabetes**					0,021
Sim	563 (36,0)	(33,6; 38,4)	262 (41,3)	(37,5; 45,1)	
Não	1.001 (64,0)	(61,6; 66,4)	373 (58,7)	(54,9; 62,5)	
**Histórico de obesidade**					<0,001
Sim	828 (52,9)	(50,5; 55,4)	238 (37,5)	(33,8; 41,3)	
Não	617 (39,5)	(37,0; 41,9)	320 (50,4)	(46,5; 54,3)	
Sem informação	119 (7,6)	(6,4; 9,0)	77 (12,1)	(9,8; 14,8)	
**Histórico de tabagismo**					>0,050
Sim	242 (15,5)	(13,7; 17,3)	100 (15,7)	(13,1; 18,7)	
Não	1.322 (84,5)	(82,7; 86,3)	535 (84,3)	(81,3; 86,9)	
Total	1.564 (100,0)	-	635 (100,0)	-	

O sexo apresentou associação com o histórico de diabetes [x^2^(1)=5,33; p-valor 0,021], mais frequente em usuários do sexo masculino (41,3%). O histórico de obesidade também apresentou associação significativa [x^2^(1)=34,69; p-valor<0,001], porém foi mais frequente em usuários do sexo feminino (52,9%). Os históricos de hipertensão e de tabagismo não apresentaram associação significativa com o sexo (p-valor>0,050).

Os valores gerais de colesterol total, colesterol da lipoproteína de baixa densidade, colesterol da lipoproteína de alta densidade e IMC foram maiores em usuários do sexo feminino. A idade e os valores da glicose, pressão arterial sistólica, pressão arterial diastólica, altura, peso e percentual de risco foram maiores em usuários do sexo masculino (Tabela 3).

**Tabela 3:** Mediana (Med) e quartis (Q1; Q3) de fatores de risco e percentual de risco entre sexos. São Leopoldo, 2018-2021 (n=2.199)

	Sexo feminino		Sexo masculino		
	Med	(Q1; Q3)	Med	(Q1; Q3)	p-valor
**Idade** (anos)	55	(46; 64)	59	(50; 65)	<0,001
**Colesterol total** (mg/dL)	193	(167; 228)	181	(153; 209)	<0,001
**LDL-ca** (mg/dL)	107	(84; 132)	102	(78; 126)	0,030
**HDL-cb** (mg/dL)	52	(44; 62)	44	(37; 51)	<0,001
**Glicose** (mg/dL)	101	(89; 133)	106	(93; 136)	0,009
**Glicemia capilar casual** (mg/dL)	157	(117; 225)	156	(119; 242)	0,983
**Hemoglobina glicada** (%)	6,5	(5,8; 10,5)	7,1	(5,9; 10,0)	0,330
**Pressão arterial sistólica** (mmHg)	130	(120; 140)	130	(120; 140)	<0,001
**Pressão arterial diastólica** (mmHg)	80	(70; 90)	80	(70; 90)	0,014
**Altura** (m)	1,56	(1,51; 1,6)	1,68	(1,63; 1,73)	<0,001
**Peso** (kg)	74,5	(64,5; 86)	80,4	(72; 92,5)	<0,001
**Índice de massa corporal** (kg/m^2^)	30,9	(26,7; 35,4)	29,1	(26,4; 32,7)	<0,001
**Percentual de risco** (%)	5,0	(3,0; 8,0)	8,0	(5,0; 11,0)	<0,001

^a^LDL-c: colesterol da lipoproteína de baixa densidade, ^b^HDL-c: colesterol da lipoproteína de alta densidade.

Após a estratificação de risco (Tabela 4), foram observadas as diferenças entre os sexos. Para os usuários de risco baixo, as variáveis colesterol da lipoproteína de alta densidade, altura, IMC, percentual de risco apresentaram diferenças significativas (p-valor<0,001), bem como a idade (p-valor 0,003). Quanto aos usuários com risco moderado, essas diferenças foram para idade, colesterol da lipoproteína de alta densidade, altura, peso (p-valor<0,001) e colesterol total (p-valor 0,001). Entre os usuários de risco alto, as diferenças concentraram-se nas variáveis idade, colesterol total, colesterol da lipoproteína de alta densidade, altura, peso (p-valor<0,001), percentual de risco (p-valor 0,004), colesterol da lipoproteína de baixa densidade (p-valor 0,025), pressão arterial sistólica (p-valor 0,010) e IMC (p-valor 0,017). Não foi possível analisar as diferenças no estrato com risco muito alto, devido ao número de participantes do sexo feminino (n=2).

**Tabela 4:** Mediana (Med) e quartis (Q1; Q3) de fatores de risco e percentual de risco por risco cardiovascular e sexo. São Leopoldo, 2018-2021 (n=2.199)

	Risco cardiovascular														
	Baixo					Moderado					Alto				
	Feminino		Masculino			Feminino		Masculino			Feminino		Masculino		
	Med	(Q1; Q3)	Med	(Q1; Q3)	p-valor	Med	(Q1; Q3)	Med	(Q1; Q3)	p-valor	Med	(Q1; Q3)	Med	(Q1; Q3)	p-valor
**Idade** (anos)	46	(38; 53)	44	(35; 50)	0,003	62	(56; 67)	58	(53; 62)	<0,001	71	(66; 75)	66	(61; 72)	<0,001
**Colesterol total** (mg/dL)	188	(160; 218)	177	(148; 214)	0,099	201	(174; 233)	185	(163; 209)	0,001	196	(173; 230)	180	(148; 204)	<0,001
**LDL-ca** (mg/dL)	105	(79; 130)	97	(70,5; 127,5)	0,529	110	(86; 136)	105	(90,5; 123,5)	0,398	110	(87; 134)	99	(69; 130)	0,025
**HDL-cb** (mg/dL)	50	(43; 59)	43	(36; 50)	<0,001	53	(46; 62)	44	(36; 52)	<0,001	56	(46; 65)	44	(38; 51)	<0,001
**Glicose** (mg/dL)	96	(86; 111)	96	(88; 102)	0,787	105	(91; 150)	109	(94; 146)	0,529	112	(95; 155)	113	(101; 161)	0,346
**Glicemia capilar casual** (mg/dL)	139	(110; 202)	142	(103; 281)	0,631	166	(122; 247)	157	(120; 253)	0,480	170	(125; 212)	153	(121; 213)	0,662
**Hemoglobina glicada** (%)	6,0	(5,7; 11,4)	6,0	(5,2; 56,05)	0,809	7,0	(5,9; 11,0)	7,0	(5,85; 9,32)	0,446	7,0	(5,85; 9,6)	7,0	(6,2; 10,0)	0,146
**Pressão arterial sistólica** (mmHg)	120	(111; 130)	120	(110; 130)	0,548	130	(120; 140)	130	(120; 140)	0,566	140	(140; 160)	140	(130; 150)	0,010
**Pressão arterial diastólica** (mmHg)	80	(70; 80)	80	(70; 80)	0,271	80	(70; 90)	80	(70; 90)	0,208	80	(80; 90)	80	(80; 90)	0,133
**Altura** (m)	1,57	(1,53; 1,62)	1,69	(1,65; 1,75)	<0,001	1,54	(1,5; 1,59)	1,67	(1,63; 1,72)	<0,001	1,52	(1,48; 1,57)	1,67	(1,62; 1,72)	<0,001
**Peso** (kg)	79	(68,5; 92,5)	80	(70,4; 92)	0,143	71	(62,2; 82)	81	(74; 93)	<0,001	70	(61; 82)	79	(70; 91)	<0,001
**Índice de massa corporal** (kg/m^2^)	31,9	(27,5; 36,7)	29,0	(25,9; 32,1)	<0,001	30,1	(26,1; 33,7)	29,2	(26,8; 33,1)	0,246	30,7	(26,5; 35,2)	29,0	(25,9; 32,6)	0,017
**Percentual de risco** (%)	3,0	(2,0; 4,0)	3,0	(2,0; 4,0)	<0,001	7,0	(5,0; 8,0)	7,0	(6,0; 8,0)	0,113	11,0	(10,0; 13,0)	12,0	(11,0; 14,0)	0,004

^a^LDL-c: colesterol da lipoproteína de baixa densidade, ^b^HDL-c: colesterol da lipoproteína de alta densidade.

O registro da prescrição de estatinas constava em 29,5% (IC95% 27,6; 31,4) dos prontuários. Estavam acima do alvo de colesterol da lipoproteína de baixa densidade 53,3% (IC95% 49,9; 56,8) dos usuários; destes, 52,1% (IC95% 47,4; 56,8) não possuíam registro da prescrição de estatina.

Houve associação significativa [x^2^(3)=83,19; p-valor<0,001] entre o registro da prescrição de estatinas e o risco cardiovascular, com maior frequência em usuários com risco alto (41,2%) (Tabela 5). Foi encontrada associação significativa [x^2^(1)=7,82; p-valor 0,005] da frequência do registro da prescrição com o alvo terapêutico de colesterol da lipoproteína de baixa densidade, com maior frequência de registro para os usuários abaixo do alvo terapêutico (51,6%). Mais da metade (51,5%; IC95% 46,4; 56,7) dos usuários acima do alvo de colesterol da lipoproteína de baixa densidade e com risco entre moderado e muito alto não possuíam registro da prescrição de estatinas.

**Tabela 5:** Frequências absoluta (n) e relativa (%) e intervalo de confiança de 95% (IC95%) do registro da prescrição de estatinas por sexo, alvo terapêutico de colesterol da lipoproteína de baixa densidade e risco cardiovascular. São Leopoldo, 2018-2021 (n=2.199)

	Prescrição de estatinas					
	Sim		Não		Total	
	n (%)	(IC95%)	n (%)	(IC95%)	n (%)	p-valor
Sexo						0,416
Feminino	453 (29,0)	(26,8; 31,2)	1.111 (71,0)	(68,8; 73,2)	1.564 (100,0)	
Masculino	195 (30,7)	(27,2; 34,4)	440 (69,3)	(65,6; 72,8)	635 (100,0)	
**Alvo terapêutico de LDL**-c^a^						0,224
Abaixo	165 (43,7)	(38,7; 48,7)	213 (56,3)	(51,3; 61,3)	378 (100,0)	
Acima	207 (47,9)	(43,2; 52,6)	225 (52,1)	(47,4; 56,8)	432 (100,0)	
**Risco cardiovascular**						<0,001
Baixo	176 (19,3)	(16,9; 22,0)	734 (80,7)	(78,0; 83,1)	910 (100,0)	
Moderado	287 (34,3)	(31,2; 37,6)	549 (65,7)	(62,4; 68,8)	836 (100,0)	
Alto	178 (41,2)	(36,6; 45,9)	254 (58,8)	(54,1; 63,4)	432 (100,0)	
Muito alto	7 (33,3)	(16,3; 54,6)	14 (66,7)	(45,4; 83,7)	21 (100,0)	

^a^LDL-c: colesterol da lipoproteína de baixa densidade.

Não foi encontrada associação (p-valor>0,050) da frequência do registro da prescrição com o sexo na amostra geral. Houve associação do registro da prescrição com o sexo ao estratificar os usuários acima do alvo de colesterol da lipoproteína de baixa densidade [x^2^(1)=4,19; p-valor 0,041], com maior frequência em usuários do sexo masculino (59,8%).

## Discussão 

Somente 54,1% dos usuários possuíam informações para classificação do risco cardiovascular, embora fosse possível utilizar o IMC em vez do colesterol total na calculadora da iniciativa HEARTS. A situação limita a robustez das análises, pode comprometer a extrapolação do perfil de risco cardiovascular encontrado e destaca a necessidade de aprimoramento dos registros, visto que esses dados subsidiam ações para redução das taxas de complicações, internações e morbimortalidade relacionadas às doenças cardiovasculares (11).

Embora o uso da calculadora da iniciativa HEARTS seja preconizada para pessoas com 40 anos ou mais, o benefício de investigar o risco cardiovascular dos usuários com idade entre 18 e 39 anos pode superar o potencial viés de classificação. Isso ocorre ao ampliar a análise do perfil da amostra e o panorama epidemiológico, alinhado ao plano de ações estratégicas para o enfrentamento das doenças crônicas e agravos não transmissíveis no Brasil no período 2021-2030 (12).

Por meio deste estudo, foi possível identificar que a maioria dos participantes apresentou risco baixo, o que pode estar relacionado à predominância do sexo feminino no perfil da amostra. Ainda que possa haver dificuldade de comparação direta com estudos prévios devido à diferença do método de cálculo, o risco cardiovascular encontrado na amostra deste estudo se assemelha ao estimado em dez anos para a população brasileira, no qual o sexo masculino e indivíduos com nenhuma ou baixa escolaridade apresentaram maior risco (19). Esse risco aumentado pode estar associado às predisposições biológicas do sexo masculino, aos hábitos de vida e às desvantagens socioeconômicas atreladas à nenhuma ou baixa escolaridade, como o acesso limitado aos serviços e às práticas de prevenção e promoção da saúde (19-21).

Apesar de a amostra ter sido composta por usuários que possuíam algum fator de risco cardiovascular, observou-se o aumento da frequência do histórico da hipertensão, diabetes e tabagismo conforme o risco. O sexo feminino apresentou maior frequência do histórico de obesidade e maiores valores de IMC, o que concorda com cenário brasileiro e regional (22-23), embora a amostra tenha apresentado maiores diferenças entre os sexos, o que pode estar relacionada à incompletude dos dados, principalmente no sexo masculino.

A associação entre o histórico de obesidade e o risco cardiovascular se apresentou de forma atípica, com maior frequência no risco baixo. É possível que isso se deva à seleção da amostra, visto que a prevalência de obesidade foi maior no sexo feminino, que compôs a maioria do estrato de risco baixo.

O histórico de diabetes foi mais frequente no sexo masculino, em contraste com a prevalência encontrada na região e no país (22,24). As variáveis pressóricas também se apresentaram maiores no sexo masculino, mesmo sem diferença na frequência do histórico de hipertensão entre os sexos. Também foi possível identificar o perfil lipídico dos usuários atendidos na Atenção Primária do município. Os valores de colesterol total e frações na amostra geral e por sexo se aproximaram ao encontrado na população adulta brasileira (25).

Não foi encontrada associação do sexo com estar ou não acima do alvo de colesterol da lipoproteína de baixa densidade. A maioria da amostra apresentou valores de colesterol da lipoproteína de baixa densidade acima do alvo. O sexo feminino apresentou maiores valores de colesterol total, colesterol da lipoproteína de alta densidade e colesterol da lipoproteína de baixa densidade. Essas diferenças podem estar relacionadas ao climatério e à menopausa, períodos relevantes para o risco cardiovascular no sexo feminino, o que reforça a necessidade do rastreio e intervenções precoces nessa população (26-27).

A frequência da prescrição de estatinas se assemelha ao encontrado na Atenção Primária de comunidades brasileiras (31,1%) (23). Neste estudo, houve associação da frequência do registro da prescrição de estatinas com o alvo terapêutico de colesterol da lipoproteína de baixa densidade. A prescrição foi mais frequente nos usuários abaixo do alvo, o que pode indicar redução do colesterol da lipoproteína de baixa densidade devido às estatinas. Porém, aproximadamente metade dos usuários acima do alvo de colesterol da lipoproteína de baixa densidade e com risco entre moderado e muito alto não possuíam registro da prescrição de estatinas. Este achado apontou possíveis barreiras no cuidado, como as dificuldades no rastreio do risco cardiovascular.

Ao mesmo tempo, houve associação da prescrição com o risco cardiovascular, mais frequente quanto maior o risco. Visto que a finalidade do uso das estatinas na prevenção primária é a diminuição do risco cardiovascular, pode-se entender que os registros das prescrições estão de acordo com a iniciativa HEARTS e com o Protocolo Clínico e Diretrizes Terapêuticas da Dislipidemia vigente no período do estudo (14-15).

Em 2025, poucos medicamentos hipolipemiantes disponibilizados no Sistema Único de Saúde podem ser adicionados ao tratamento para beneficiar usuários de mais alto risco que não atingiram o alvo de colesterol da lipoproteína de baixa densidade somente com o uso de estatinas (14). A prescrição de sinvastatina predomina em comparação à de atorvastatina, possivelmente devido ao tipo de receituário necessário e ao custo dos medicamentos, o que pode dificultar o acesso ao tratamento otimizado (28).

Apesar da disponibilização gratuita de medicamentos para o controle de diabetes, hipertensão e dislipidemia no Sistema Único de Saúde, as políticas de prevenção primária ainda são insuficientes para o controle dos fatores de risco cardiovascular (23).

Os resultados deste estudo forneceram um panorama sobre ações de redução do risco cardiovascular por meio de estatinas. A ausência de análise dos dados sobre qual estatina foi prescrita e sobre a alimentação e a atividade física pode ter limitado potenciais inferências do estudo. 

O rastreio precoce, o controle medicamentoso, a atividade física, a alimentação saudável e a cessação do tabagismo são fundamentais no manejo do risco cardiovascular. Ainda assim, barreiras de acesso aos serviços de saúde dificultam a adesão e o acompanhamento, o que pode acarretar complicações associadas (15,20-21). A baixa prevalência de saúde cardiovascular ideal na população brasileira destaca a disparidade entre o cuidado prestado e o ideal (29). Em vista disso, emerge a desigualdade entre a prestação de assistência e de cuidados, comparativamente ao ideal projetado, o que evidencia lacuna na eficácia da atuação da equipe multiprofissional.

Para qualificar a atenção integral na prevenção e o controle das doenças cardiovasculares, os planos de ação dos municípios devem abordar a prevenção e promoção da saúde, a capacitação de profissionais e gestores, o diagnóstico precoce das morbidades, o fortalecimento da gestão e da infraestrutura e o estímulo à pesquisa e inovação (11). Devem incluir como prioridade ações de educação permanente, a fim de sensibilizar os profissionais sobre o tema e, ao mesmo tempo, promover ações de alimentação adequada e saudável, aconselhamento de atividades físicas e enfrentamento ao uso do tabaco, juntamente com a articulação intersetorial para produção social da saúde através da promoção do desenvolvimento sustentável (30).

Os resultados reforçam a necessidade de planejar outras estratégias de rastreamento do risco e de prevenção de eventos cardiovasculares na Atenção Primária de São Leopoldo. A maioria dos usuários apresentou risco cardiovascular entre baixo e moderado e a prescrição de estatinas alcançou menos de um terço da amostra geral, embora estivesse alinhada com as diretrizes ao considerar o risco na tomada de decisão.
